# The vascular-cambium-specific transcription factor PtrSCZ1 and its homologue regulate cambium activity and affect xylem development in *Populus trichocarpa*

**DOI:** 10.3389/fpls.2025.1546660

**Published:** 2025-03-11

**Authors:** Yi Sun, Jianing Jiang, Qiongyue Zhang, Jinfeng Zhao, Hongyan Ma, Danning Li, Shuang Li, Chenguang Zhou, Wei Li

**Affiliations:** State Key Laboratory of Tree Genetics and Breeding, Northeast Forestry University, Harbin, China

**Keywords:** vascular cambium, xylem development, wood formation, transcription factor, PtrSCZs, *Populus trichocarpa*

## Abstract

**Introduction:**

Vascular cambium proliferates and differentiates into the secondary xylem (wood), enabling the perennial increase in stem diameter for wood formation. In our previous study, we identified 95 vascular-cambium-specific (VCS) transcription factors (TFs) in *Populus trichocarpa*.

**Methods:**

In this study, we characterized the function of the highly vascular cambium-expressed heat shock TF among these VCSs, PtrSCZ1, using *PtrSCZ1*-overexpressing transgenic lines and gene-edited mutants in *P. trichocarpa*.

**Results:**

Overexpressing *PtrSCZ1* or its homolog *PtrSCZ3* (*OE-PtrSCZ1*, *OE-PtrSCZ3*) led to enhanced cambium activity, increased stem diameter, and a larger xylem proportion. CRISPR-based mutants of *PtrSCZ1* and *PtrSCZ3* exhibited phenotypes opposite to the *OE-PtrSCZ1* and *OE-PtrSCZ3* plants. This suggests that *PtrSCZ1* and *PtrSCZ3* redundantly promote cambium activity and secondary growth, leading to increased radial growth in *P. trichocarpa*. Overexpression and knockout of *PtrSCZ1* and *PtrSCZ3* significantly affected the expression of key regulatory factors of cambium (*PtrWOX4a*, *PtrWOX4b*, *PtrWOX13a*, *PtrPXYa*, *PtrVCM1*, and *PtrVCM2*) and disrupted cell wall-related gene expression. This demonstrates that *PtrSCZ1* and *PtrSCZ3* may function in cambium division activity by regulating these key cambium-associated transcription factors for wood formation.

**Discussion:**

Our work identifies *PtrSCZ1* and *PtrSCZ3* as promising target genes for enhancing wood yield through molecular breeding, and illustrates the role of vascular cambium systems in understanding lateral meristem development.

## 1 Introduction

Vascular tissue formation in forest trees initiates from the procambium within the shoot apical meristems (SAM). This tissue differentiates into vascular bundles while maintaining a fascicular cambium layer that retains meristematic potential. The fascicular cambium, together with the interfascicular cambium derived from parenchyma cells, forms a continuous ring of secondary meristematic tissue, collectively referred to as the vascular cambium (Mazur et al., 2014; Zhu et al., 2018, 2019). The vascular cambium continuously produces secondary xylem inward and secondary phloem outward during secondary growth (Timell, 1980; Włoch, 1981; Włoch et al., 2023; Li et al., 2024). Fusiform initials differentiate into vessels and fibers, contributing to increased stem diameter and wood formation (Esau, 1965; Evert, 2006). Therefore, the activity of the vascular cambium directly affects the secondary growth of trees and, consequently, wood formation. Cambium activity is tightly regulated by transcription factors (TFs), plant hormones, signaling peptides, small RNAs, long non-coding RNAs, and enzymes. These elements contribute to transcriptional, post-transcriptional, and epigenetic regulation according to developmental programs (Ortega-Martínez et al., 2007; Matsumoto-Kitano et al., 2008; Suer et al., 2011; Nieminen et al., 2015; Johnsson and Fischer, 2016; Bhalerao and Fischer, 2017; Fischer et al., 2019; Zhang et al., 2019b; Hu et al., 2024). However, the mechanisms governing this complex regulatory process remain largely unknown.

Several TFs expressed in the stem cambium have been identified in tree species. These include WOX4a/b, PXY, PtrVCM1 and PtrVCM2, PtrWOX13a, PtrVCS2, PtrHB4, PtrHB7, popREVOLUTA (PRE), ARK2, POPCORONA, PtoTCP20, PagGRF15, PagMYB31, and PagJAZ5. They have been shown to directly or indirectly regulate cambium activity (Du et al., 2009, 2011; Robischon et al., 2011; Zhu et al., 2013; Kucukoglu et al., 2017; Zhu et al., 2018; Hou et al., 2020; Zheng et al., 2021; Dai et al., 2023; Hu et al., 2024; Zhang et al., 2024; Zhou et al., 2024). Disturbances in the expression levels of these genes affect the number of cambium cell layers, subsequently influencing xylem development. This indicates their crucial roles in regulating vascular cambium activity and xylem development.

Heat shock transcription factors (HSFs) are crucial transcriptional regulators involved in early heat shock responses (Li et al., 2010; Nishizawa-Yokoi et al., 2011; Ikeda et al., 2011) and disease resistance (Kumar et al., 2009). Based on structural characteristics, such as the adjacent hydrophobic heptad repeat oligomerization domain (HR-A/B), plant HSFs can be grouped into three conserved classes (Nover et al., 2001; Scharf et al., 2012). AtHSFB4/SCHIZORIZA (SCZ), a B-class heat-shock TF, is one of the 21 TFs in the *Arabidopsis* HSF family (Nover et al., 2001; Pernas et al., 2010). The expression of *SCZ* is barely affected by heat treatment, unlike typical HSF genes such as *HSFA1s*, *HSFA2*, *HSFB1*, and *HSFB2b* (Miller and Mittler, 2006; von-Koskull-Döring et al., 2007; Scharf et al., 2012; Pardal et al., 2023). This suggests that SCZ has evolved toward functions beyond the stress response typical of the HSF family (Pardal et al., 2023). During plant development, SCZ is essential for the early establishment of stem cells that generate ground tissue in the embryonic root meristem (Pernas et al., 2010). This transcription factor also plays a key role in regulating segregation of different root tissue fates in *Arabidopsis* (Pardal et al., 2023). Overexpressing *AtHSFB4* causes abnormal growth in root meristematic region (Begum et al., 2013), while the *AtHsfB4* mutant exhibits aberrant periclinal division of the ground tissue during embryogenesis, disturbed asymmetric cell division, abnormal root hair development, and misexpression of tissue identity markers in root development (Mylona et al., 2002; ten Hove et al., 2010; Pardal et al., 2023).

Thirty HSF members have been identified in *P. trichocarpa* (Wang et al., 2011; Scharf et al., 2012; Zhang et al., 2015; Liu et al., 2019; Guo et al., 2023). PtrHSFB3-1 is a tension wood-responsive TF that directly regulates cell-wall genes, including *PAL2*, *PAL4*, *PAL5*, *C4H1*, *C3H3*, *CSE1*, *CCoAOMT2*, and *CCR2*, to reprogram wood formation (Liu et al., 2021). While some HSF genes are involved in secondary growth, most play crucial roles in stress response processes in trees. For instance, *PuHSFA4a* regulates abiotic stress responses and root development-related genes, enhancing tolerance to excess Zn in *P. ussuriensis* roots (Zhang et al., 2019a). Additionally, *PsnHSF21* in poplar contributes to salt resistance (Guo et al., 2023).

In our previous study, we identified 95 vascular-cambium-specific (VCS) TFs in *P. trichocarpa* (Dai et al., 2023). Among the 95 VCS TFs, three belonged to the HSF family: PtrVCS9, PtrVCS38, and PtrVCS80. Notably, *PtrVCS9* exhibited higher transcript abundance in the vascular cambium compared to xylem and phloem, and its expression level ranked 9th among the 95 TFs (Dai et al., 2023). In this study, we investigated the function of PtrVCS9 (renamed as PtrSCZ1) in regulating vascular cambium activity during wood formation in *P. trichocarpa*. We demonstrated that PtrVCS9 and its homologous PtrVCS80 (renamed as PtrSCZ3) are crucial for regulating vascular cambium proliferation, which in turn influences xylem cell division during secondary stem growth in *P. trichocarpa*. The two TFs regulate the expression of key genes in the stem vascular cambium, thereby modulating wood formation.

## 2 Materials and methods

### 2.1 Plant materials and growth conditions

*Populus trichocarpa* (Nisqually-1) was cultivated in a phytotron under a 16-hour light/8-hour dark photoperiod at 25°C and utilized for gene cloning and expression analysis in this study. Following generation, screening, and verification of transgenic plants, they were maintained in the phytotron for 4 months before undergoing detailed characterization.

### 2.2 Gene cloning and sequence analysis

Based on the *P. trichocarpa* genome (https://phytozome-next.jgi.doe.gov), the full-length sequences of *PtrSCZ1* and *PtrSCZ3* were amplified from cDNA derived from cambium–phloem tissue of *P. trichocarpa* plants. The specific primers used for amplification are listed in **Supplementary Table S1**. The resulting amplicons were cloned into pENTR/D-TOPO vector (Invitrogen), and their sequences were subsequently verified by sequencing. Sequence alignment was performed using Clustal W, and the corresponding figure was generated using ESPript 3.0 (Robert and Gouet, 2014). Phylogenetic analysis of SCZs was conducted using MEGA 5 software, employing the neighbor-joining method with 1000 bootstrap replicates.

### 2.3 Total RNA extraction

Well-developed plants were selected for bark removal. Stem-differentiating xylem (SDX) from the debarked stems and cambium–phloem tissues from the bark side were separately collected using single-edged razors and immediately frozen in liquid nitrogen. Total RNA was isolated from SDX tissues using the RNeasy Plant Mini Kit (Qiagen, 74904) for RT-qPCR analysis of lignin biosynthesis-related genes. For cambium–phloem tissues, RNA extraction was performed using the CTAB method (Lorenz et al., 2010) to determine the relative expression levels of *PtrSCZ1*, *PtrSCZ3*, and other cambium-related genes. RNA concentration was quantified using a NanoDrop 2000 spectrophotometer (Thermo Scientific).

### 2.4 RT-qPCR

Reverse transcription (RT) reactions were performed using TaqMan Reverse Transcription Reagents (Invitrogen, N8080234) according to the manufacturer’s protocol. All quantitative PCR (qPCR) analyses were conducted on the Agilent M×3000P Real-Time PCR System with FastStart Universal SYBR Green Master Mix (Roche, 4913914001) following the standard protocol. Gene expression levels were normalized to *PtrACTIN*. The specific primers used for RT-qPCR analysis are listed in **Supplementary Table S1**.

### 2.5 *In situ* RNA localization

The eighth stem internodes of *P. trichocarpa* were collected and fixed in FAA solution (50% [v/v] ethanol, 5% [v/v] acetic acid, and 3.7% [v/v] formaldehyde). Following dehydration, the fixed tissues were embedded in paraffin (Sigma) and sectioned to a thickness of 10 µm using a rotary microtome (Leica RM2245). A 172-bp region of *PtrSCZ1* was used as a specific probe for *in situ* hybridization. Both antisense and sense probes were synthesized using T7 and SP6 RNA polymerases, respectively. Probe labeling was performed using a digoxigenin RNA labeling kit (Roche). After pretreatment, the slide-mounted sections were hybridized with the digoxigenin-labeled *PtrSCZ1* antisense or sense RNA probes in hybridization solution. Hybridized signals were detected by incubation with alkaline phosphatase-conjugated anti-digoxigenin antibodies according to the manufacturer’s protocol provided with the digoxigenin nucleic acid detection kit (Roche). Color development was achieved using alkaline phosphatase substrates. Microscopic images were captured using a Leica DM6B microscope. The Primer sequences used for probe amplification are summarized in **Supplementary Table S1**.

### 2.6 Constructs generation and genetic transformation

The *35S::PtrSCZ1-FLAG* and *35S::PtrSCZ3-FLAG* constructs were generated using the pBI121 vector. Double-knockout mutants of *PtrSCZ1* and *PtrSCZ3* genes were created using the CRISPR-Cas9 system. The single guide RNAs (sgRNAs) were designed using CRISPR-P 2.0 (http://crispr.hzau.edu.cn/cgi-bin/CRISPR2/CRISPR). The sgRNA sequences were synthesized and subsequently cloned into the pEgP237-2A GFP vector (Ueta et al., 2017). All vectors were introduced into *P. trichocarpa* plants through *Agrobacterium tumefaciens*-mediated transformation, following a previously described protocol (Li et al., 2017). The expression levels of *PtrSCZ1* and *PtrSCZ3* in transgenic plants were analyzed by RT-qPCR as described above. To verify the genome editing in knockout mutants, specific primers flanking the sgRNA target sites were designed for PCR amplification. The resulting PCR products were cloned into the pMD18-T vector, and a minimum of 20 colonies were selected for sequencing analysis. All primer sequence are listed in **Supplementary Table S1**.

### 2.7 Histological analysis

Stem internodes of *P. trichocarpa* were sectioned into 2-mm fragments and fixed in FAA solution (composition as described above). The fixed stem segments were subsequently processed through a graded ethanol series (50%, 60%, 70%, 85%, and 100% [v/v]) at 4°C for dehydration, followed by incubated in ethanol:xylene solutions (75:25, 50:50, and 25:75 [v/v]) and finally in 100% xylene at room temperature. The dehydrated samples were then immersed in 75:25 (v/v) xylene:paraffin solution at 42°C overnight and embedded in pure paraffin (Sigma). The paraffin-embedded stem segments were sectioned into 12-µm thickness using a rotary microtome (Leica RM2245) and stained with Toluidine Blue. Digital images were acquired using a M8 microscope scanner (Percipient), and the quantitative measurements of cambium cells, stem area, and xylem area were performed using LASv4.8 and LASXv2.0 software (Leica).

### 2.8 Wood chemistry

Stem segments from *OE-PtrSCZ1*-L14, *OE-PtrSCZ3*-L13, *ptrscz1/ptrscz3*-L1, and wild-type *P. trichocarpa* plants were ground to 40–60 mesh size. Extraction was carried out using a benzene:ethanol mixture (2:1 v/v) for 24 hours. The air-dried wood samples from different transgenic lines and wild-type plants were pooled, and their lignin and sugar contents were analyzed according to established procedures (Abraham et al., 2013; Wang et al., 2018).

### 2.9 Statistical analysis

All statistical analyses were performed using two-tailed Student’s *t*-tests to determine significance, with significance levels set at *P < 0.05 and **P < 0.01. The RNA-seq data for *PtrSCZ1* (*PtrVCS9*) and *PtrSCZ3* (*PtrVCS80*) presented in this study were obtained from previously characterized vascular-cambium-specific TFs. These TFs were identified through RNA-seq analysis of laser capture microdissection (LCM)-isolated cambium, differentiating xylem, and phloem tissues (**Supplementary Table 1** in Dai et al., 2023), integrated with RNA-seq data from leaves, roots, and shoots (Shi et al., 2017).

## 3 Results

### 3.1 *PtrSCZ1* abundantly expressed in the vascular cambium of *P. trichocarpa*

**Figure 1:**
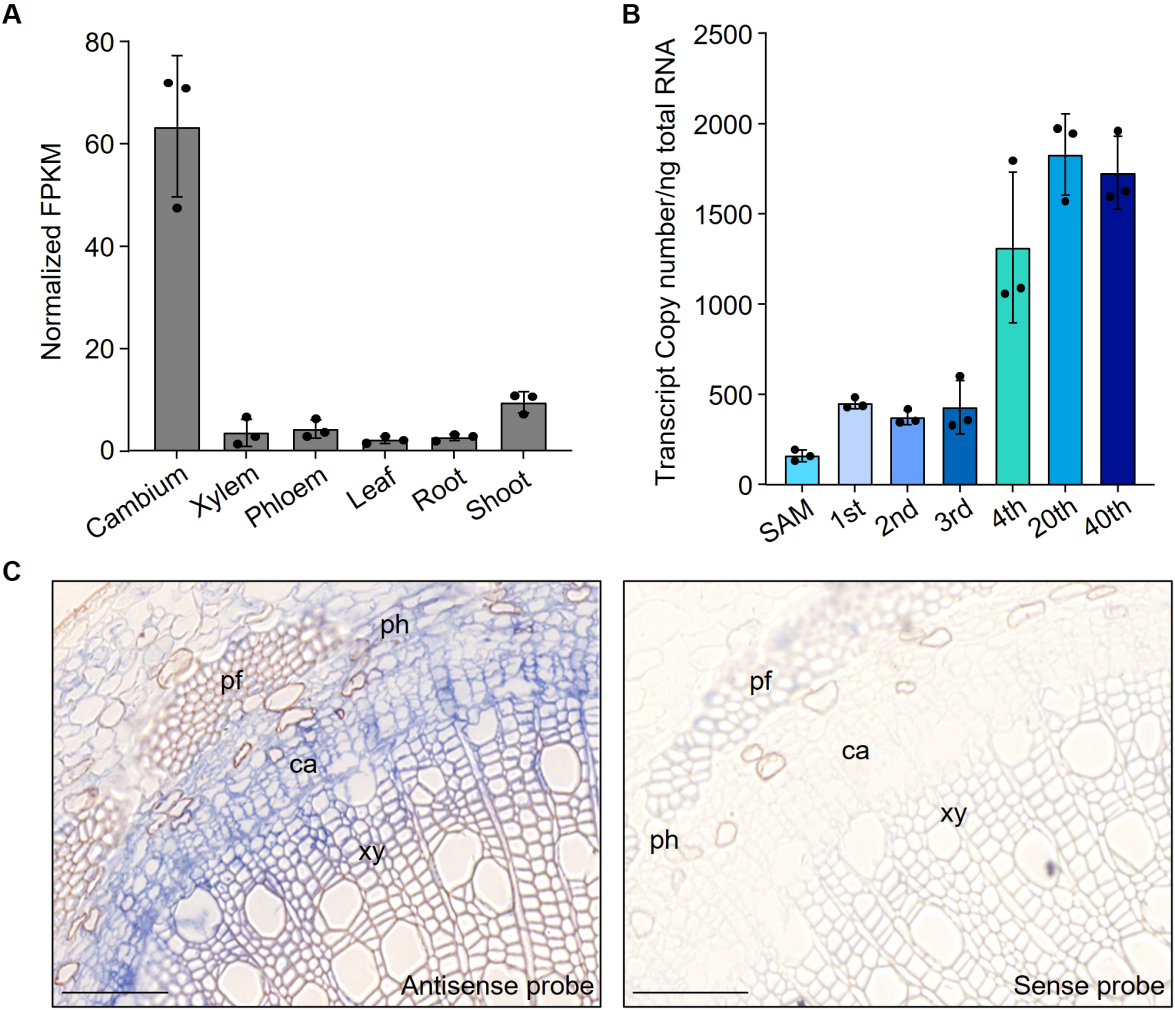
*PtrSCZ1* was highly expressed in vascular cambium. **(A)** Tissue-specific expression patterns of *PtrSCZ1* analyzed by RNA-seq across six *P. trichocarpa* tissues (cambium, differentiating xylem, phloem, leaf, root, and shoot). FPKM values represent fragments per kilobase of transcript per million fragments mapped reads. **(B)** Transcript abundance of *PtrSCZ1*, as determined by RT-qPCR in the shoot apices (containing the apical meristem, leaf primordia, developing leaves and early vascular tissues), and cambium–phloem tissues from 1st–4th, 20th, and 40th internodes of *P. trichocarpa* stems. **(C)** Spatial expression pattern of *PtrSCZ1* revealed by *in situ* hybridization in 8th stem internode of *P. trichocarpa*. Stem cross-sections were hybridized with digoxigenin-labeled antisense RNA probes for *PtrSCZ1* (left) or sense RNA probes as negative control (right). ca, cambium; pf, phloem fiber; ph, phloem; xy, xylem. Bar, 100 µm. Error bars in **(A, B)** represent standard errors (SE) of three biological replicates from independent pools of *P. trichocarpa* tissues.

Based on vascular-cambium-specific transcriptome data acquired by LCM (Dai et al., 2023) and tissue-specific datasets (Shi et al., 2017) from *P. trichocarpa*, we identified that the HSF gene *PtrVCS9* shows preferential expression in the vascular cambium compared to other tissues including xylem, phloem, leaf, root, and shoot (**Figure 1A**). Comparative analysis with *Arabidopsis* HSF family genes revealed that PtrVCS9 shares sequence similarity with HSFB4 (**Supplementary Figure S1**), also known as SCZ (SCHIZORIZA) (Pernas et al., 2010; ten Hove et al., 2010). Consequently, we designated PtrVCS9 as PtrSCZ1. RT-qPCR analysis demonstrated that *PtrSCZ1* expression in the cambium becomes evident beginning at the 4th internode, coinciding with the initiation of secondary growth (**Figure 1B**). *In situ* RNA hybridization using a *PtrSCZ1*-specific probe on 4-month-old *P. trichocarpa* 8th internodes showed strong signal accumulation in the vascular cambium zone and weaker signals in xylem cells (**Figure 1C**). The expression pattern suggests PtrSCZ1’s involvement in vascular cambium development. To explore PtrSCZ1’s functions in cambium activity, we generated CRISPR-mediated knockout and overexpressing lines in *P. trichocarpa* for functional characterizations.

### 3.2 PtrSCZ1 positively regulates cambium activity

**Figure 2:**
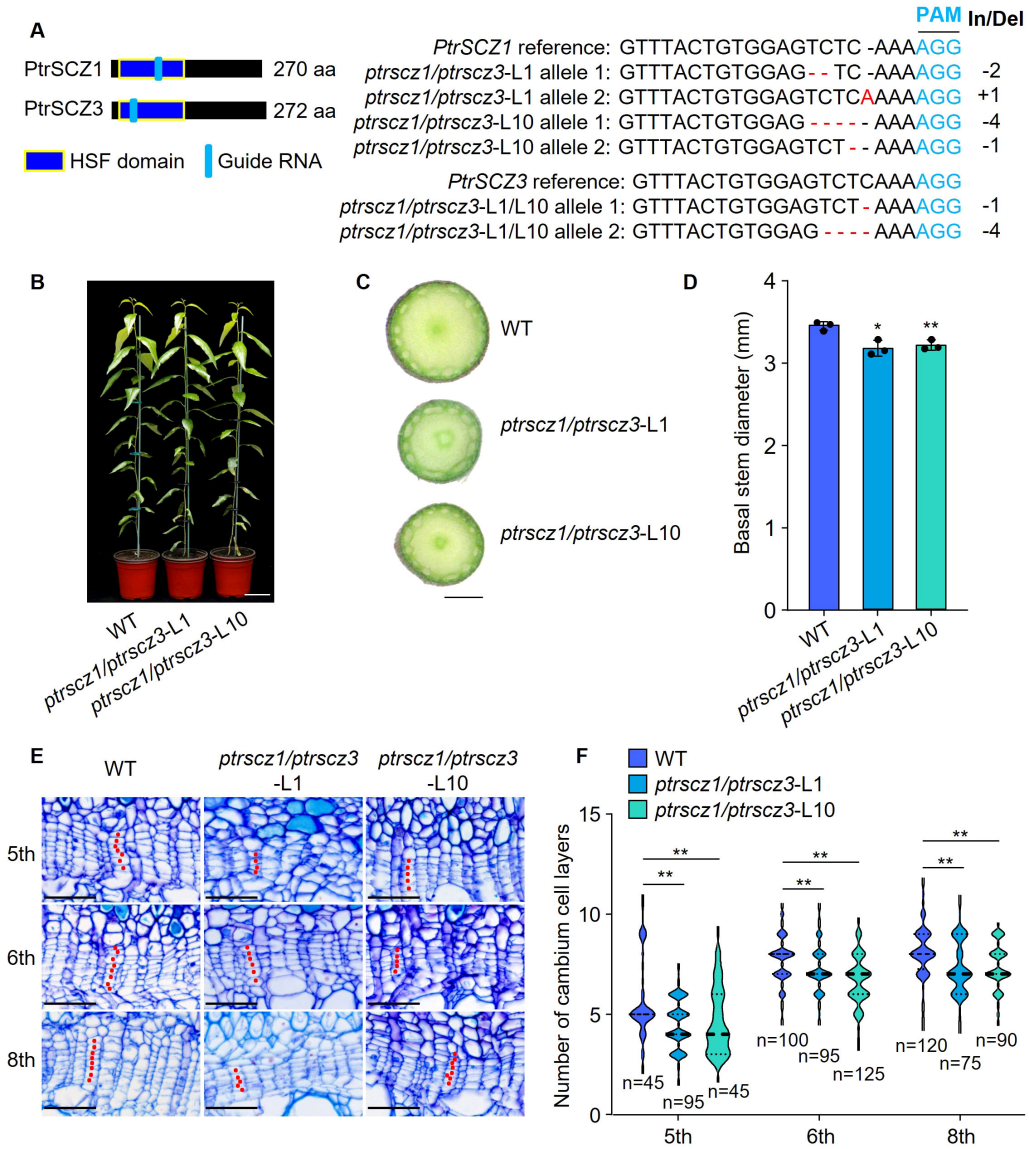
Effects of knocking out *PtrSCZ1* and *PtrSCZ3* on vascular cambium proliferation. **(A)** Mutations at the sgRNA target sites in *PtrSCZ1* and *PtrSCZ3* from two independent *ptrscz1/ptrscz3* mutant lines. Deletions are indicated by red dashes, while nucleotide substitutions and insertions are highlighted in red. The protospacer-adjacent motif (PAM) is shown in blue, with insertion/deletion (In/Del) lengths indicated on the right. **(B)** Growth phenotypes of 4-month-old WT, *ptrscz1/ptrscz3-*L1, and *ptrscz1/ptrscz3-*L10 plants. Bar, 10 cm. **(C)** Basal stems morphology of WT, *ptrscz1/ptrscz3-*L1, and *ptrscz1/ptrscz3-*L10 mutant plants. Bar, 1 mm. **(D)** Quantitative analysis of stem diameters of WT, *ptrscz1/ptrscz3-*L1, and *ptrscz1/ptrscz3-*L10 plants. Data represent means ± SE from three biological replicates (two-tailed Student’s *t*-test, *P< 0.05, **P< 0.01). **(E)** Histochemical and histological characterization of stem sections from WT, *ptrscz1/ptrscz3-*L1, and *ptrscz1/ptrscz3-*L10 plants. Bars, 50 µm. **(F)** Cambium cell layer quantification in stem vascular tissues of WT, *ptrscz1/ptrscz3-*L1, and *ptrscz1/ptrscz3-*L10 plants. For each biological replicate, at least ten radial cell files were analyzed per cross-section. Data from three biological replicates are presented (two-tailed Student’s *t*-test, **P< 0.01). n, total number of samples per dataset. The width of the violin represents the distribution shape of the data. The center line represents the median, and the upper and lower dotted lines correspond to the 25th and 75th percentiles, respectively.

We generated loss-of-function mutants of *PtrSCZ1* in *P. trichocarpa* using CRISPR-based genome editing with *Streptococcus pyogenes* Cas9 (Deltcheva et al., 2011; Jiang et al., 2013; Heler et al., 2015). The single-knockout *ptrscz1* lines showed comparable plant height, stem diameter, and cambium cell layer numbers to wild-type (WT) plants (**Supplementary Figure S2**), suggesting potential functional redundancy among *PtrSCZ* family members. We therefore investigated *PtrSCZ1’s* homologue, *PtrSCZ3*, which shares 93.3% amino acid sequence identity with PtrSCZ1 (**Supplementary Figure S1**) and displays high expression levels in cambium, root, and shoot tissues (**Supplementary Figure S3A**) (Shi et al., 2017; Dai et al., 2023), though lacking cambium-specific expression across different internodes (**Supplementary Figure S3B**). *In situ* hybridization demonstrated that *PtrSCZ3* exhibits a comparable expression pattern to *PtrSCZ1* in stem tissues (**Supplementary Figures S3C, D**). We subsequently created two independent biallelic double mutants, *ptrscz1/ptrscz3-*L1 and *ptrscz1/ptrscz3*-L10, containing insertions and/or deletions that introduce frameshifts and premature stop codons (**Figures 2A, B**). The *ptrscz1/ptrscz3* lines displayed significantly reduced stem diameters compared to WT plants (**Figures 2C, D**; **Supplementary Figure S4**). Stem cross-sectional analysis revealed that *ptrscz1/ptrscz3* internodes consistently lacked a fixed number (one to three) of cambium cell layers compared to the WT (5th to 8th internodes shown in **Figures 2E, F**). These results indicate that the loss-of-function in PtrSCZ1 and PtrSCZ3 intrinsically reduces cambium cell layer formation during stem elongation.

### 3.3 Overexpressing *PtrSCZ1* or *PtrSCZ3* produced effects opposite to their respective loss-of-function mutations in *P. trichocarpa*

**Figure 3:**
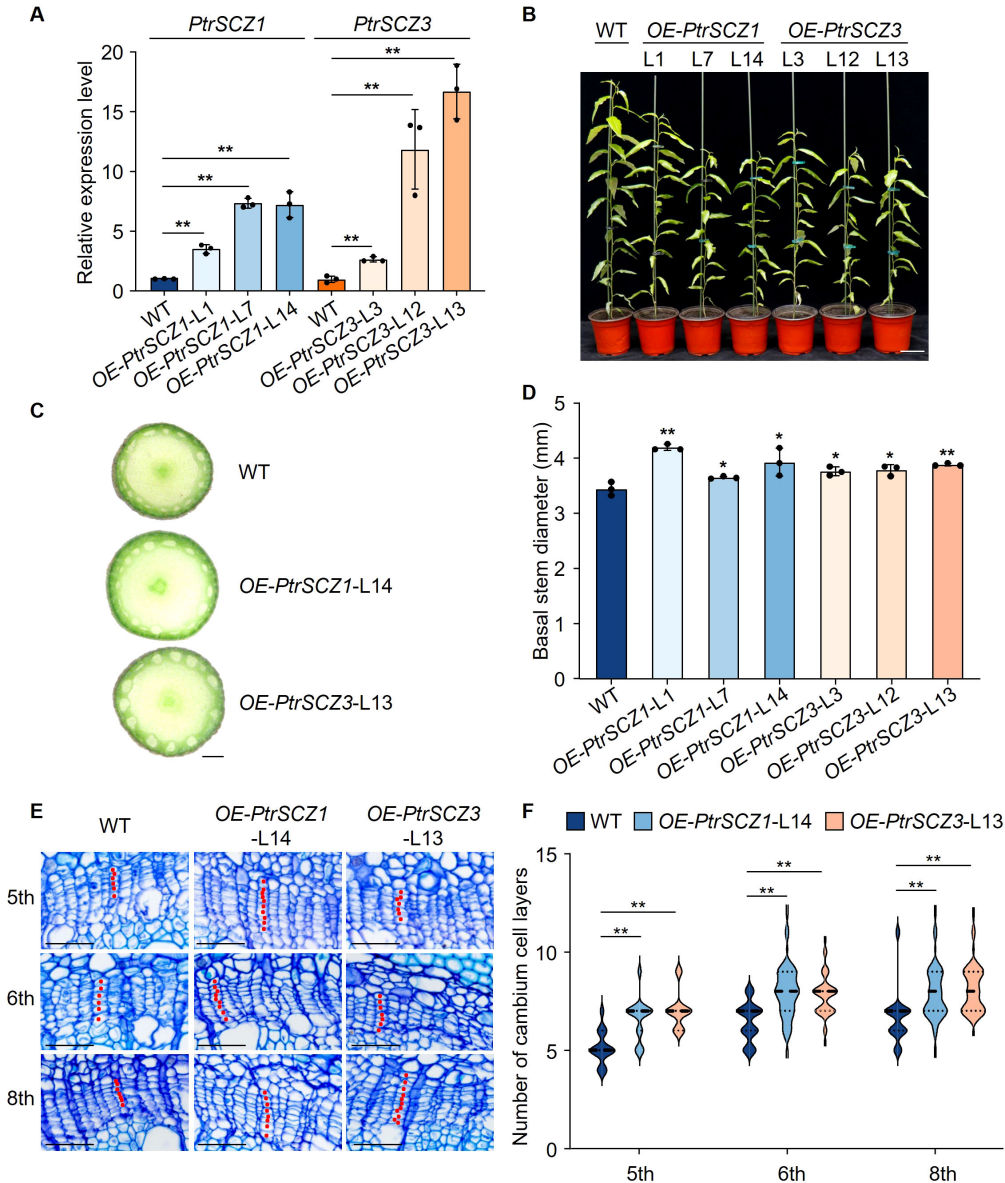
Growth phenotype and statistical analysis of *PtrSCZ1* and *PtrSCZ3* overexpression transgenic lines. **(A)** Expression profiles of *PtrSCZ1* and *PtrSCZ3* in cambium–phloem tissues from transgenic lines. **(B)** Growth phenotypes of 4-month-old WT, *OE-PtrSCZ1* (-L1, -L7, -L14), and *OE-PtrSCZ3* (-L3, -L12, -L13) plants. Bar, 10 cm. **(C)** Basal stem morphology of WT, *OE-PtrSCZ1*-L14, and *OE-PtrSCZ3*-L13 transgenic plants. Bar, 1 mm. **(D)** Quantitative analysis of stem diameters in WT, *OE-PtrSCZ1* (-L1, -L7, -L14), and *OE-PtrSCZ3* (-L3, -L12, -L13) plants. In **(A, D)**, error bars represent mean ± SE from three biological replicates, with three *P. trichocarpa* plants per genotype per replicate (two-tailed Student’s *t*-test, *P< 0.05, **P<0.01). **(E)** Histochemical and histological characterization of stem sections from WT, *OE-PtrSCZ1-*L14, and *OE-PtrSCZ3-*L13 transgenic plants. Bars, 50 µm. **(F)** Cambium cell layer quantification in stem vascular tissues of WT, *OE-PtrSCZ1*-L14, and *OE-PtrSCZ3*-L13 transgenic plants. For each biological replicate, at least ten radial cell files were counted per cross-section. Data from three biological replicates are shown (two-tailed Student’s *t*-test, **P< 0.01). n = 30 in **(F)**. The width of the violin represents the distribution shape of the data. The center line represents the median, and the upper and lower dotted lines correspond to the 25th and 75th percentiles, respectively.

We overexpressed *PtrSCZ1* in *P. trichocarpa* under the control of a cauliflower mosaic virus 35S promoter. Three independent overexpression transgenic lines of *OE-PtrSCZ1* (-L1, -L7 and -L14) were generated (**Figures 3A, B**). These transgenic lines displayed stunted growth (**Figure 3B**) but showed increased stem diameter during secondary development compared to WT (**Figures 3C, D**; **Supplementary Figure S5**). To examine PtrSCZ1’s role in cambium development during wood formation, we selected *OE-PtrSCZ1-*L14, which showed the highest transgene overexpression level, for detailed phenotypic analysis. Four-month-old transgenic plants were evaluated under greenhouse conditions. Histological examination revealed that *OE-PtrSCZ1*-L14 developed 2–3 additional cambium cell layers in the 5th to 8th stem internodes compared to WT (**Figures 3E, F**). Comparable phenotypic alterations were observed in *OE-PtrSCZ1*-L13 (**Figures 3E, F**). The opposing cambium cell layer phenotypes between gain- and loss-of-function transgenics indicate that *PtrSCZ1* and *PtrSCZ3* play specific roles in regulating cambium cell proliferation.

### 3.4 *PtrSCZ1* and *PtrSCZ3* regulate cambium activity by altering the expression of key TFs involved in cambium development

**Figure 4:**
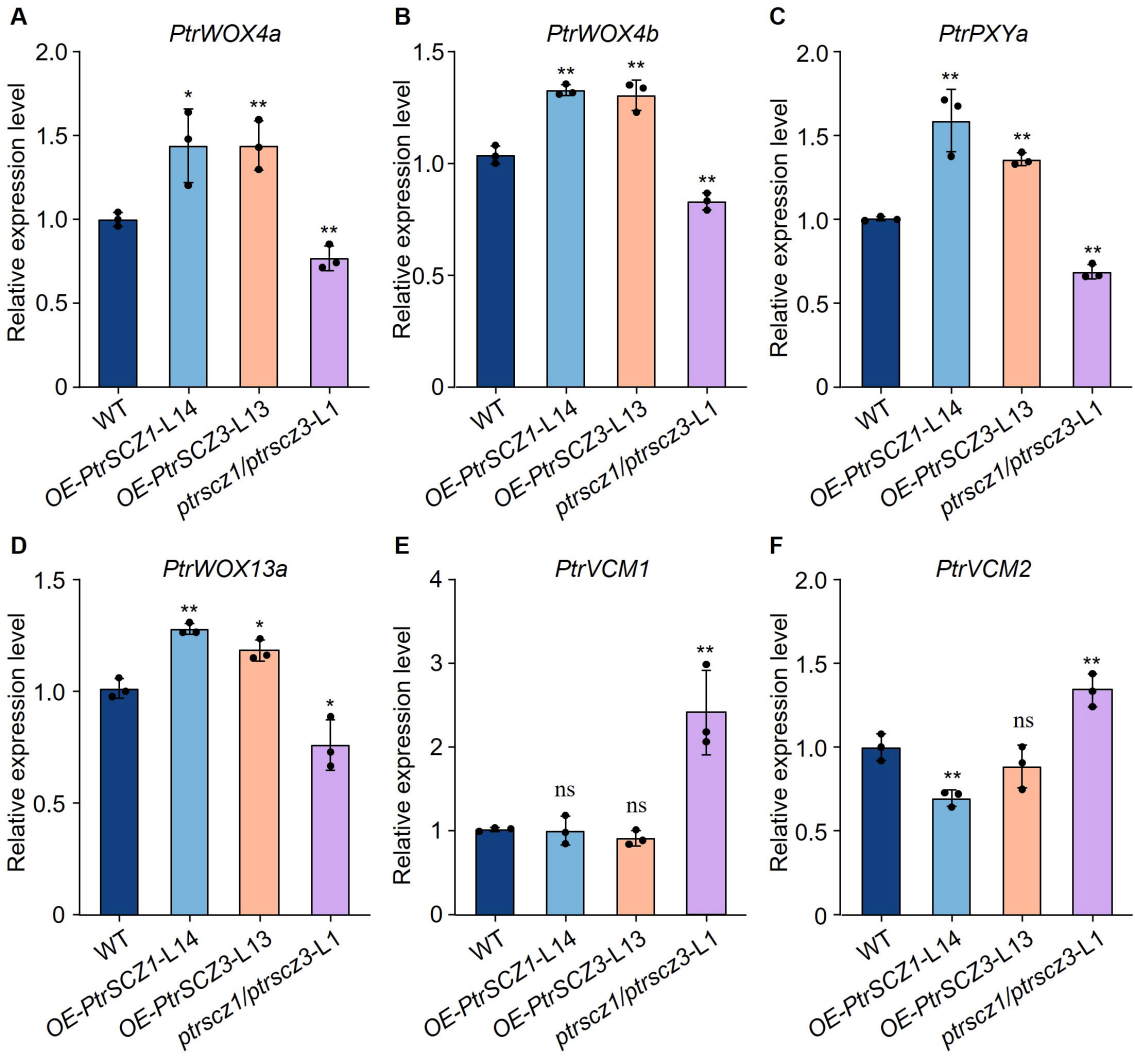
RT-qPCR analysis of key TFs in cambium development in transgenic plants. **(A–F)** Expression levels of *PtrWOX4a* **(A)**, *PtrWOX4b* **(B)**, *PtrPXYa* **(C)**, *PtrWOX13a* **(D)**, *PtrVCM1* **(E)**, and *PtrVCM2* **(F)** were quantified by RT-qPCR in cambium–phloem tissues from 4-month-old WT, *OE-PtrSCZ1*-L14, *OE-PtrSCZ3*-L13, and *ptrscz1/scz3*-L1 transgenic plants. Error bars represent mean ± SE from three biological replicates using independent pools of *P. trichocarpa* cambium–phloem tissues (two-tailed Student’s *t*-test, *P< 0.05, **P< 0.01). ns, not significant differences.

The *ptrscz1/ptrscz3* mutants displayed reduced cambium cell layer numbers compared to WT plants, whereas overexpression transgenic plants showed increased cambial cell layers (**Figures 2**, **3**). These results indicate that *PtrSCZ1* and *PtrSCZ3* positively regulate cambial activity. To investigate how alterations in *PtrSCZ1* and *PtrSCZ3* affect cambial development, we analyzed the expression levels of key cambium regulators, including positive regulators of cell division (*PtrWOX4a*, *PtrWOX4b*, *PtrWOX13a*, and *PtrPXYa*) and negative regulators of vascular cambium proliferation (*PtrVCM1* and *PtrVCM2*) in *OE-PtrSCZ1*, *OE-PtrSCZ3*, and *ptrscz1/ptrscz3* plants (Etchells et al., 2013; Kucukoglu et al., 2017; Zheng et al., 2021; Dai et al., 2023). Transcript levels of *PtrWOX4a*, *PtrWOX4b*, *PtrWOX13a*, and *PtrPXYa* were consistently upregulated in *OE-PtrSCZ1* and *OE-PtrSCZ3* plants but downregulated in *ptrscz1/ptrscz3* mutants compared to WT (**Figures 4A–D**). *PtrVCM2* expression was significantly reduced in *OE-PtrSCZ1* plants, while *PtrVCM1* levels remained unchanged in both *OE-PtrSCZ1* and *OE-PtrSCZ3* lines (**Figures 4E, F**). Most key TFs associated with cambium activity showed altered expression patterns in response to *PtrSCZ1* and *PtrSCZ3* manipulation. These findings suggest that PtrSCZ1 and PtrSCZ3 may promote cambium cell division by modulating these key TFs.

### 3.5 Elevated levels of *PtrSCZ1* and *PtrSCZ3* transcripts significantly impact xylem development, as well as lignin contents in stem wood

**Figure 5:**
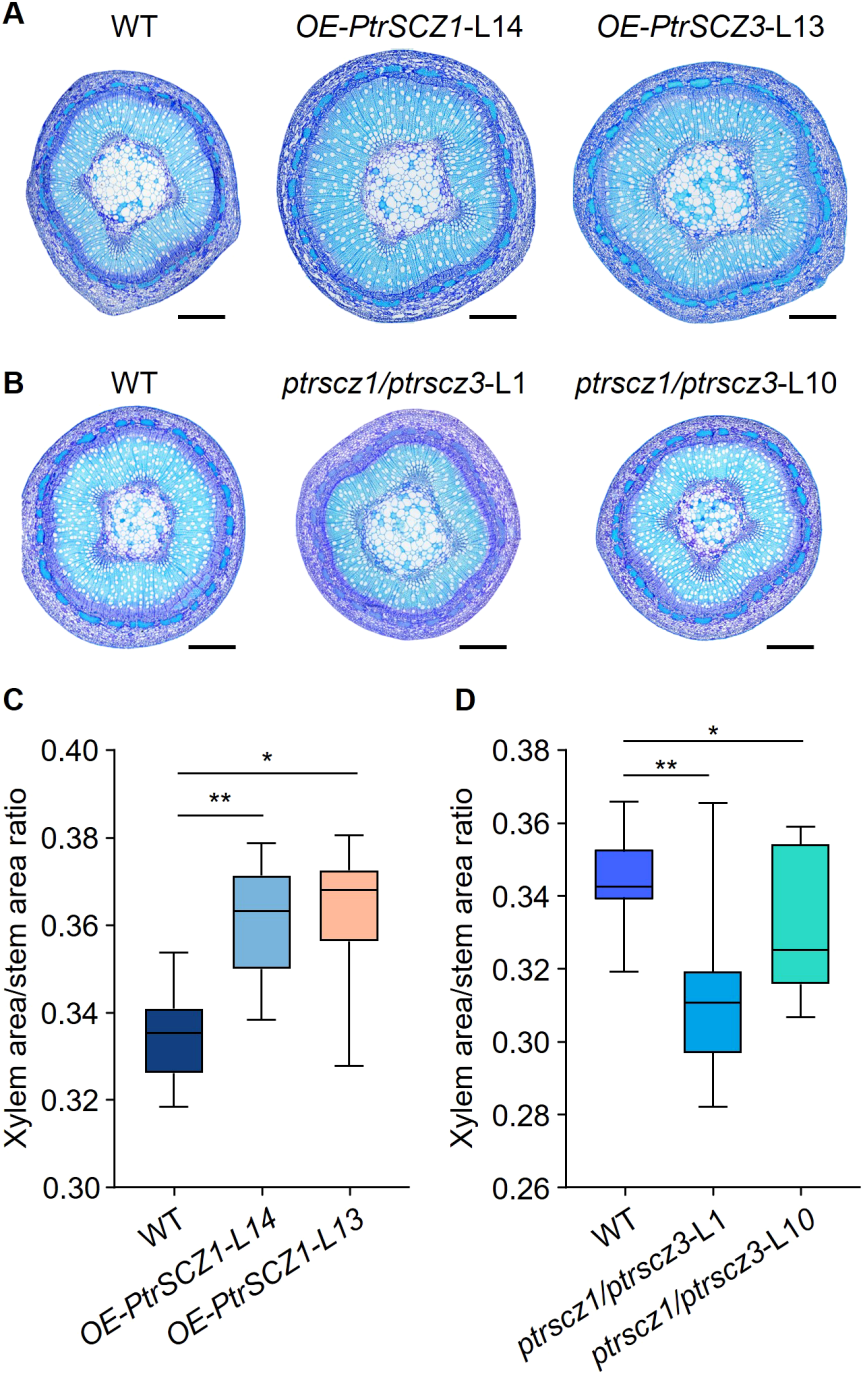
*PtrSCZ1* and *PtrSCZ3* affect the xylem development. **(A, B)** Histological observations of stem cross-sections from 8th internodes of WT, *OE-PtrSCZ1*-L14, *OE-PtrSCZ3*-L13, *ptrscz1/ptrscz3*-L1, and *ptrscz1/ptrscz3*-L10 plants. Bars, 500 µm. **(C, D)** Quantitative analysis of xylem area proportion in stems among *OE-PtrSCZ1*-L14, *OE-PtrSCZ3*-L13, and WT **(C)**, *ptrscz1/ptrscz3*-L1, *ptrscz1/ptrscz3*-L10, and WT **(D)**. Xylem and stem areas were measured from five cross-sections per stem internode for each biological replicate. Data from three biological replicates are shown (two-tailed Student’s *t*-test, *P< 0.05, **P < 0.01). n=15. Box plots display median and interquartile ranges, with whiskers representing data ranges excluding outliers.

To determine whether cambium cell layer alterations affect xylem development, we compared xylem zones between transgenic and WT plants. Stem cross-sectional analysis revealed that *OE-PtrSCZ1-*L14 and *OE-PtrSCZ3-*L13 transgenic lines displayed increased xylem-to-stem area ratios compared to WT (**Figures 5A, C**; **Supplementary Figures S6A, B)**, demonstrating enhanced xylem development in the overexpression plants. In contrast, *ptrscz1/ptrscz3* loss-of-function mutants showed significantly reduced xylem area proportions relative to total stem area (**Figures 5B, D**; **Supplementary Figures S6C, D**), indicating inhibited xylem development. These findings demonstrate that *PtrSCZ1* and *PtrSCZ3* positively regulates secondary growth. We subsequently characterized and quantified the three primary components of wood cell walls.

Wood composition analysis showed a notable increase in lignin content (13%–15%) in *OE-PtrSCZ1-*L14 and *OE-PtrSCZ3*-L13 (**Table 1**), with no significant change in total carbohydrate content in these transgenic plants. Notably, monolignol composition in the lignin of overexpression transgenics showed a 10% reduction in S-monomers and a 20% increase in G-monomers, leading to S:G ratios of 1.48–1.62 compared to 2.00 in WT (**Table 2**). Analytical results showed reduced lignin content (approximately 9%) and the carbohydrate-to-lignin ratio (C:L) in *ptrscz1/ptrscz3-*L1 mutants, contrasting with the increases observed in *OE-PtrSCZ1*-L14 and *OE-PtrSCZ3*-L13 (**Table 1**). S:G ratios in *ptrscz1/ptrscz3*-L1 mutants showed minimal changes, remaining statistically indistinguishable from WT values (**Table 2**). These findings suggest that *PtrSCZ1* and *PtrSCZ3* regulate lignin biosynthesis without substantially affecting lignin monomer composition. We subsequently examined the effect of *PtrSCZ1* and *PtrSCZ3* overexpression or mutation on the transcriptional levels of genes related to monolignol biosynthesis.

**Table 1:** Wood composition of transgenic and wild-type *P. trichocarpa*.

Plants	WT	*OE-PtrSCZ1* -L14	*OE-PtrSCZ3* -L13	*ptrscz1/ptrscz3* -L1
Total Lignin	21.18 ± 0.58	24.26 ± 0.89*	24.02 ± 0.40*	19.29 ± 0.30*
Acid-Insoluble	18.26 ± 0.33	20.90 ± 1.10	20.26 ± 0.31*	17.02 ± 0.31
Acid-soluble	2.92 ± 0.25	3.35 ± 0.22	3.76 ± 0.11*	2.28 ± 0.01
Glucose	47.26 ± 1.46	49.13 ± 0.88	51.23 ± 0.90	44.94 ± 0.72
Xylose	13.93 ± 1.69	17.92 ± 1.35	14.90 ± 0.20	14.68 ± 0.19
Galactose	4.82 ± 0.65	3.33 ± 0.19	4.49 ± 0.61	6.68 ± 0.22
Arabinose	1.84 ± 0.49	2.02 ± 0.52	2.33 ± 0.48	3.80 ± 0.13*
Total Carbohydrate	67.84 ± 2.22	72.41 ± 0.75	72.96 ± 0.57	70.11 ± 1.14
C:L	3.21 ± 0.15	2.98 ± 0.08	3.04 ± 0.05	3.77 ± 0.04*

Units: g/100g of dry extractive-free wood. C:L=Carbohydrate to Lignin Ratio. Four-month-old plants were tested. Data are mean of three independent assays. *P<0.05.

**Table 2:** Lignin composition of transgenic and wild-type *P. trichocarpa*.

Plants	WT	*OE-PtrSCZ1*-L14	*OE-PtrSCZ3*-L13	*ptrscz1/ptrscz3*-L1
S-Lignin	61.54% ± 0.04%	56.68% ± 1.54%*	55.61% ± 1.19%*	64.32% ± 0.7%
G-Lignin	30.81% ± 0.01%	34.97% ± 0.97%*	37.01% ± 1.14%**	30.69% ± 0.67%
H-Lignin	7.65% ± 0.01%	8.35% ± 0.58%	6.94% ± 0.24%	4.99% ± 0.34%
S/G ratio	2.00 ± 0.09	1.62 ± 0.09 *	1.48 ± 0.08*	2.10 ± 0.07

H, H-subunits; G, G-subunits; S, S-subunits; %, percentage volume of total lignin. Four-month-old plants were tested. Data are mean of three independent assays. *P<0.05, **P<0.01.

### 3.6 PtrSCZ1 and PtrSCZ3 regulate monolignol biosynthesis

**Figure 6:**
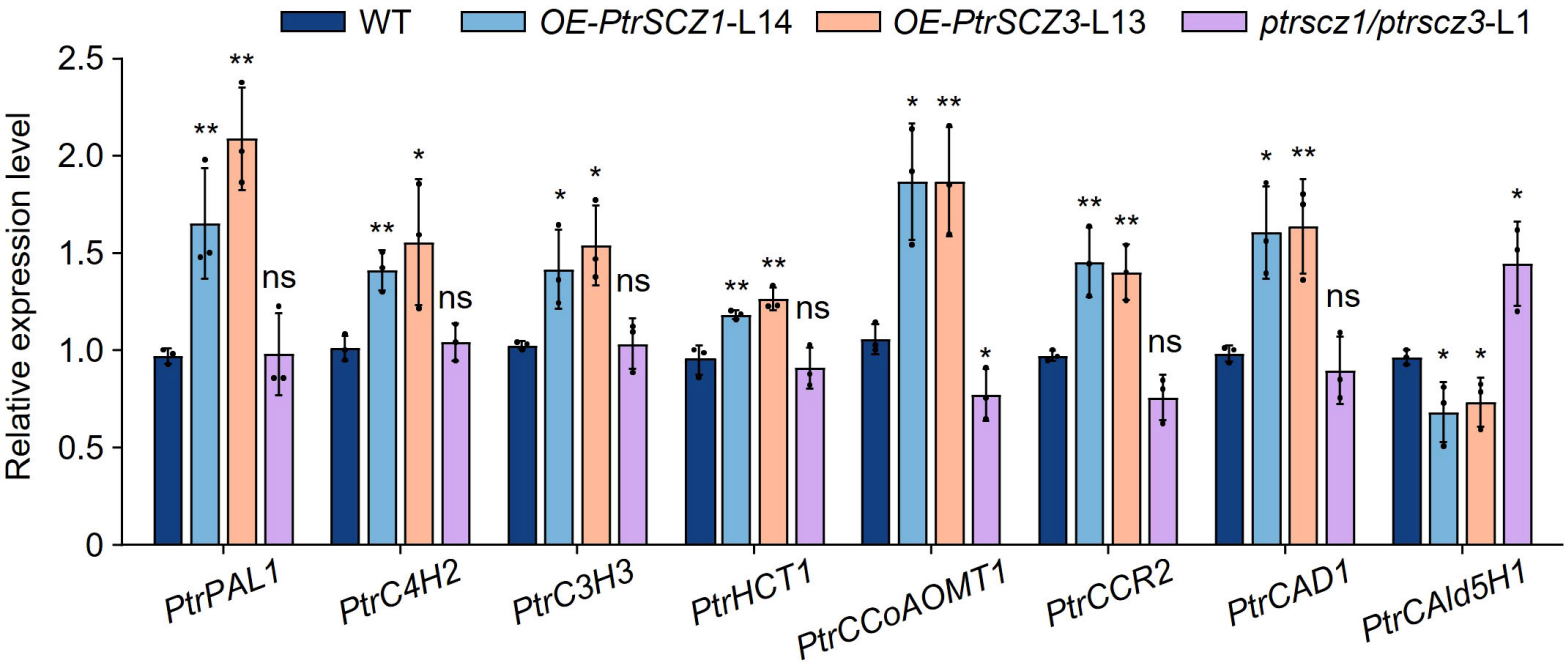
Expression levels of cell wall component genes in transgenic plants. The SDX tissues from WT, *OE-PtrSCZ1*-L14, *OE-PtrSCZ3-*L13, and *ptrscz1/ptrscz3*-L1 plants were analyzed by RT-qPCR to examine expression patterns of monolignol biosynthesis genes. Genes are arranged according to their enzymatic order in the lignin biosynthesis pathway. Error bars represent mean ± SE from three biological replicates using independent pools of *P. trichocarpa* SDX tissues (two-tailed Student’s *t*-test, *P< 0.05, **P< 0.01). ns, not significant differences.

Given that altering *PtrSCZ1* and *PtrSCZ3* functions resulted in changes in lignin content and S/G ratio, we use RT-qPCR to examine the expression of monolignol biosynthesis enzyme genes in transgenic plants. Enzyme genes associated with the G-lignin biosynthesis (Zhang et al., 2020), including *PtrCCoAOMT1*, as well as those regulating total lignin content (*PtrPAL1*, *PtrCCR2*, and *PtrCAD1*), showed upregulated expression in both *OE-PtrSCZ1* and *OE-PtrSCZ3* (**Figure 6**). Conversely, *PtrCAld5H1* expression was downregulated (**Figure 6**), consistent with the reduced S-monomer levels observed in *OE-PtrSCZ1*-L14 and *OE-PtrSCZ3*-L13 (**Table 2**). In *ptrscz1/ptrscz3*-L1 mutants, disruption of *PtrSCZ1* and *PtrSCZ3* did not significantly alter the expression of most lignin biosynthesis genes, except for *PtrCCoAOMT1* and *PtrCAld5H1* (**Figure 6**; **Supplementary Figure S7**). Compared to overexpressed plants, these two genes displayed opposite expression patterns in the mutant (**Figure 6**). These results suggest that PtrSCZ1 and PtrSCZ3 likely function as transcriptional activators regulating lignin biosynthesis during secondary wall formation in *P. trichocarpa.*

Collectively, transcriptional analysis of *PtrSCZ1* and *PtrSCZ3* knockout and overexpressed plants reveals their active role in secondary growth through directly or indirectly regulating the vascular cambium and monolignol genes, which are essential for the cambium and xylem development.

## 4 Discussion

Division and differentiation of vascular cambium cells result in secondary xylem formation, a sequential developmental process that includes secondary cell wall (SCW) deposition and programmed cell death (Du and Groover, 2010; Luo and Li, 2022). In this study, we revealed the homologous gene pair *PtrSCZ1* and *PtrSCZ3* function redundantly in the regulation of vascular cambium activity and secondary xylem tissue formation in *P. trichocarpa* stems. We speculate that *PtrSCZ1* and *PtrSCZ3* may control wood formation by direct or indirect regulating specific vascular cambium and monolignol genes during plant secondary growth.

A key characteristic of perennial trees is the secondary growth of stems, which depends on vascular cambium proliferation activity. The expression level of *PtrSCZ1* is significantly upregulated in the vascular cambium during the secondary growth stage (**Figure 1B**). Overexpression of *PtrSCZ1* and its homologous *PtrSCZ3* promoted vascular cambium proliferation and enhanced the secondary growth of stems. The double mutants of *PtrSCZ1* and *PtrSCZ3* exhibit decreased stem diameter (**Figures 2C, D**), reduced number of vascular cambium cell layers (**Figures 2E, F**), and a reduction in the proportion of xylem (**Figures 5B, D**). The observed transgenic phenotypes demonstrate that *PtrSCZ1* and its homologs positively regulate cambium activity and xylem development. There are 4 homologous *HSFB4* genes in *P. trichocarpa* including *PtrSCZ1*, *PtrSCZ2*, *PtrSCZ3*, and *PtrSCZ4* (**Supplementary Figure S1**). Both *PtrSCZ2* (*PtrVCS38*) and *PtrSCZ4* show expression in multiple tissues, including cambium, xylem, phloem, leaves, roots, and shoots (Shi et al., 2017; Dai et al., 2023). Notably, *PtrSCZ2* has been identified as a cambium-specific TF gene (Dai et al., 2023). The potential roles of *PtrSCZ2* and *PtrSCZ4* in regulating cambium activity and affecting xylem development remain to be investigated. Generating and analyzing a quadruple mutant (*ptrscz1/ptrscz2/ptrscz3/ptrscz4*) could provide further insights into the collective functions of *PtrSCZ* genes in regulating secondary growth in woody trees.

The activity of the vascular cambium is indicated by the number of cambium cell layers, which is controlled by the expression levels of key cambium-regulated TFs. Overexpression of *PttPXY* and *PttCLE41* in hybrid aspen resulted in vascular tissue abnormalities and poor plant growth (Etchells et al., 2015; Kucukoglu et al., 2017). The *PttWOX4* genes have been shown to function downstream of *PXY* and control cell division activity in the vascular cambium, thereby influencing growth in stem girth (Kucukoglu et al., 2017). *PtrWOX13a* directly regulates the transcription of *PtrWOX4a* (Dai et al., 2023). The hierarchical regulation of cambium development is precisely controlled by these TFs. Changes in the expression levels of *PtrSCZ1* and *PtrSCZ3* were positively correlated with those of four genes (*PtrWOX4a*, *PtrWOX4b*, *PtrWOX13a*, and *PtrPXYa*) (**Figure 4**), indicating that *PtrSCZ1* and *PtrSCZ3* might operate within the same regulatory pathway, directly or indirectly influencing their expression. Knockdown of two vascular cambium-related MADS-box genes, *VCM1* and *VCM2*, enhanced cambium proliferation and subsequent xylem differentiation in *Populus* (Zheng et al., 2021). Changes in the expression of *PtrSCZ1* and *PtrSCZ3* also affect the expression of these two genes (**Figure 4**). The previous report indicated that the complex PtrWOX13a–PtrVCS2–PtrGCN5-1–PtrADA2b-3 binds directly to *PtrWOX4a* promoter through PtrWOX13a, where PtrVCS2 prevented the interaction between PtrGCN5-1 and PtrADA2b-3. This prevention resulted in *PtrWOX4a* promoter hypoacetylation, leading to fewer cambium cell layers (Dai et al., 2023). Overexpressing *PtrSCZ1* or *PtrSCZ3* elevates *PtrWOX4a* levels, yet their impact on the acetylation of the PtrWOX13a–PtrVCS2–PtrGCN5-1–PtrADA2b-3 complex remains unexplored. These findings underscore the crucial role of *PtrSCZ1* and *PtrSCZ3* in cambium activity and suggest they might establish a complex regulatory network with key TFs for cambium regulation. High levels of *PtrSCZ1* and *PtrSCZ3* may signal a regulatory cascade impacting xylem radial width, consistent with their expression in developing xylem (**Figure 1C**; **Supplementary Figure S3C**).

RT-qPCR assays showed that the monolignol genes *PtrPAL1*, *PtrC4H2*, *PtrC3H3*, *PtrHCT1*, *PtrCCoAOMT1*, *PtrCCR2*, and *PtrCAD1* were upregulated in the differentiating xylem of *OE-PtrSCZ1*-L14 and *OE-PtrSCZ3*-L13 plants (**Figure 6**). However, mutations in *PtrSCZ1* and *PtrSCZ3* did not significantly alter the expression of most monolignol genes, except for *PtrCCoAOMT1* and *PtrCAld5H1* (**Figure 6**; **Supplementary Figure S7**). This might be due to PtrSCZ1 and PtrSCZ3 functioning as direct regulators of cambium development rather than as controllers of cell wall components. The observed increase in total lignin content in *OE-PtrSCZ1* and *OE-PtrSCZ3* (**Table 1**) might be attributed to an indirect effect of the larger xylem area (**Figures 5A, C**; **Supplementary Figure S6A**). Another possibility is that changes in lignin biosynthesis gene expression in the overexpressed plants (**Figure 6**) could be due to the ectopic expression of *PtrSCZ1* and *PtrSCZ3*.

Similarly, plants with overexpressed *PtrSCZ1* and *PtrSCZ3* were dwarfed (**Figure 3**), while those with loss-of-function did not exhibit changes in height (**Figure 2**), likely due to ectopic overexpression of these genes. Considering the crucial roles of phytohormones in plant growth and development, regulations may be altered in transgenics with ectopic expression of *PtrSCZ1* and *PtrSCZ3* (Vanstraelen and Benková, 2012; Vargas et al., 2016; Hu et al., 2024). *PtrSCZ1* and *PtrSCZ3* might interact with a receptor protein perceived with phytohormones, disrupting a signaling pathway essential for releasing hormones that support normal plant growth and development. High levels of *PtrSCZ1* and *PtrSCZ3* may inhibit the hormone release, resulting in impeded growth.

## 5 Conclusion

In this study, we characterized *PtrSCZ1*, a previously uncharacterized HSF gene exhibiting specific expression in the vascular cambium. Overexpression of *PtrSCZ1* and its homolog *PtrSCZ3* significantly enhanced vascular cambial proliferation, resulting in increased stem radial growth during secondary growth. The *ptrscz1/ptrscz3* double mutants displayed phenotypes opposite to those observed in the overexpression plants. Subsequent RT-qPCR analysis of key TFs regulating cambium development in transgenic plants demonstrated that *PtrSCZ1* and *PtrSCZ3* promote cambium activity through directly or indirectly regulation of these factors, establishing a complex regulatory network controlling cambial division and xylem development. These findings identify *PtrSCZ1* and *PtrSCZ3* as promising target genes for molecular breeding strategies aimed at enhancing wood production. Moreover, this work establishes vascular cambium systems as valuable models for investigating lateral meristem development, particularly wood formation mechanisms.

## Data Availability

The datasets presented in this study can be found in online repositories. The names of the repository/repositories and accession number(s) can be found in the article/**Supplementary Material**.
