# Supplementary material for: The vascular-cambium-specific transcription factor PtrSCZ1 and its homologue regulate cambium activity and affect xylem development in Populus trichocarpa
Source: Front Plant Sci. 2025 Mar 11;16:1546660. doi: 10.3389/fpls.2025.1546660 (PMC11933121; doi:10.3389/fpls.2025.1546660)
Supplement: Supplementary file 1 [file Image1.pdf]

A

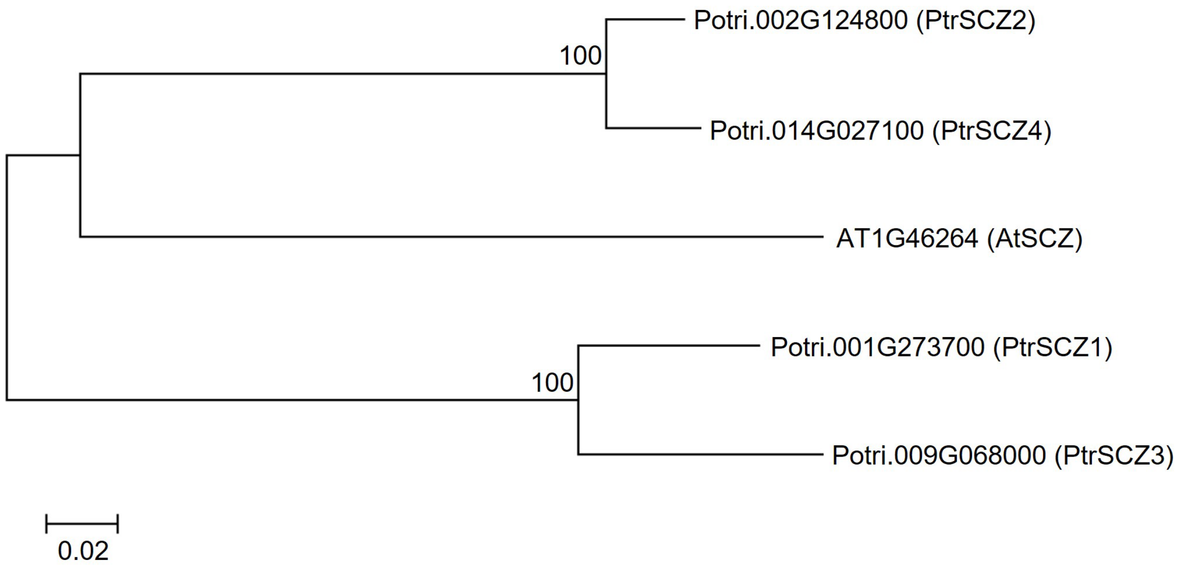

B

Family	TFs name	Identity	Similarity
HSF4B	PtrSCZ1		
	PtrSCZ2	49.7%	76.8%
	PtrSCZ3	86.9%	93.3%
	PtrSCZ4	50%	76.8%

Supplementary Figure 1. Protein sequence alignment of PtrSCZ1 and PtrSCZ3. (A) Phylogenetic relationships among PtrSCZ1 and its homologs in *P. trichocarpa* and *Arabidopsis*. The phylogenetic tree was generated using MEGA7 software with neighbor-joining algorithm and 1000 bootstrap replicates. Bar, 0.02 amino acid substitutions per site. **(B)** Sequence comparison between PtrSCZ1 and PtrSCZ3 proteins, showing 86.9% sequence identity and 93.3% sequence similarity. Multiple sequence alignment was performed using ClustalW.

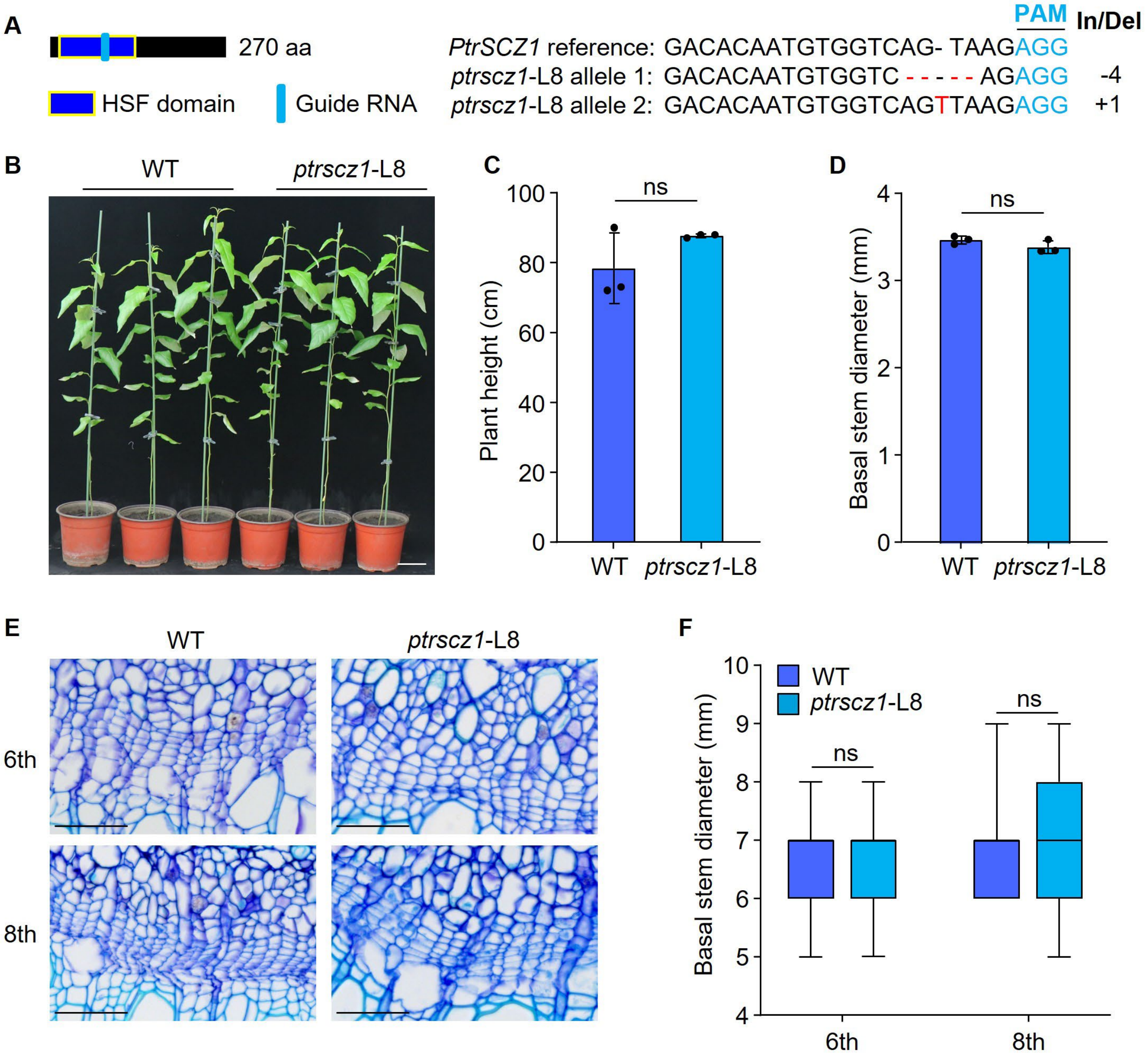

Supplementary Figure 2. Effects of single knocking out *PtrSCZ1* on vascular cambium proliferation. (A) Mutations at the sgRNA target sites in *PtrSCZ1* from *ptrscz1* mutant lines. Nucleotide deletions are indicated by red dashes, while substitutions and insertions are highlighted in red. The protospacer-adjacent motif (PAM) is shown in blue, with insertion/deletion (In/Del) lengths indicated on the right. **(B)** Growth phenotypes of 4-month-old wild-type (WT) and *ptrscz1*-L8 plants. Bar, 10 cm. **(C, D)** Quantitative analysis of plant height (C) and basal stem diameter (D) in WT and *ptrscz1*-L8 plants. **(E)** Histochemistry and histological characterization of stem sections from WT and *ptrscz1*-L8 plants. Bars, 50 µm. **(F)** Cambium cell layer quantification in stem vascular tissues of WT and *ptrscz1*-L8 plants. For each biological replicate, at least ten radial cell files were analyzed per cross-section. Data from three biological replicates are presented. Two-tailed Student's *t*-test. ns, not significant differences. Box plots display median and interquartile ranges, with whiskers representing data ranges excluding outliers.

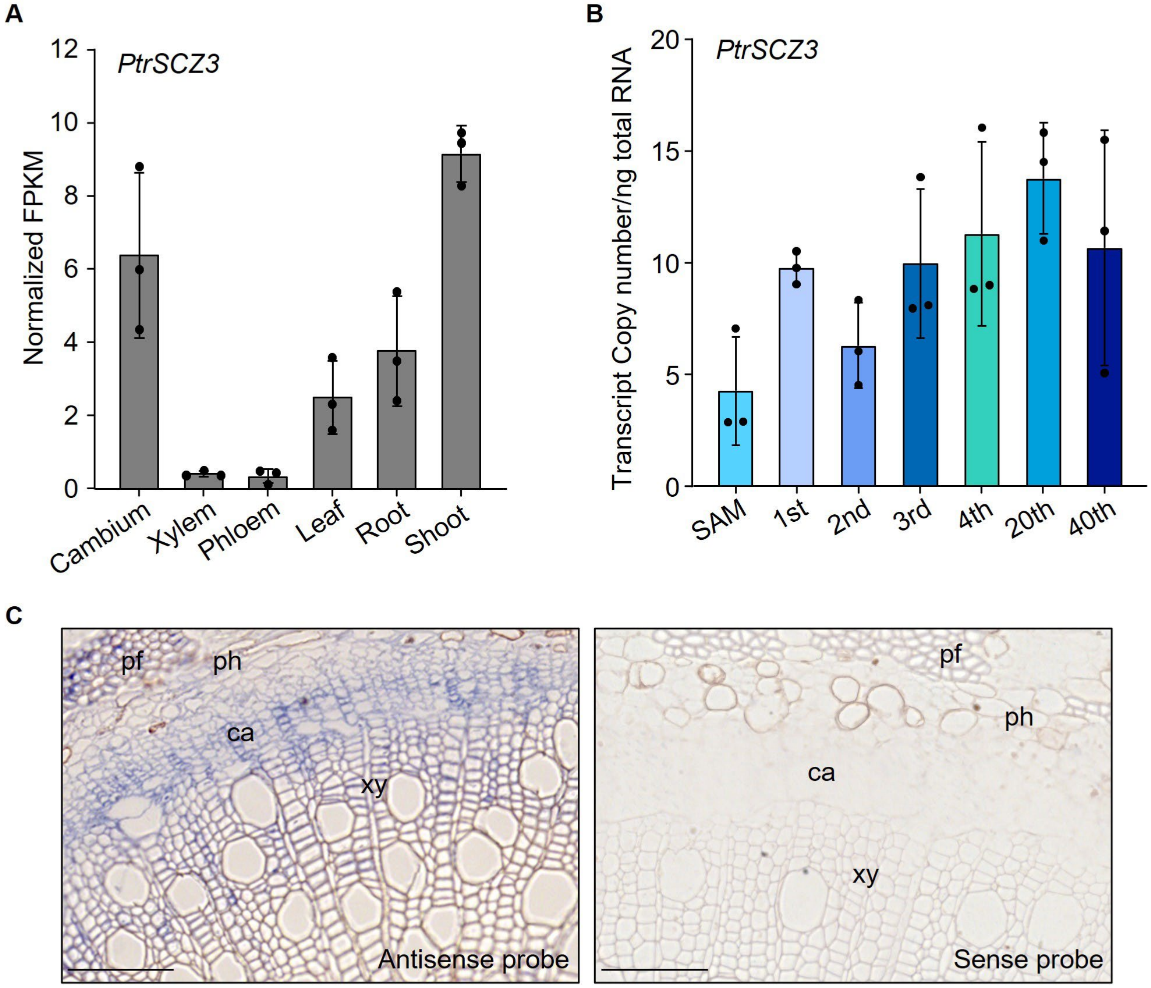

Supplementary Figure 3. Expression patterns of *PtrSCZ3*. (A) Tissue-specific expression patterns of *PtrSCZ3* analyzed by RNA-seq across six tissues (cambium, differentiating xylem, phloem, leaf, root, and shoot) of *P. trichocarpa*. FPKM values represent fragments per kilobase of transcript per million mapped reads. **(B)** Developmental expression patterns of *PtrSCZ3* determined by RT-qPCR in shoot apices (containing the apical meristem, leaf primordia, developing leaves and early vascular tissues), and cambium–phloem tissues from 1st–4th, 20th and 40th internodes of *P. trichocarpa* stems. Error bars in (A) and (B) represent mean ± SE from three biological replicates using independent pools of *P. trichocarpa* tissues. **(C)** Spatial expression pattern of *PtrSCZ3* revealed by in situ hybridization in 8th stem internodes of *P. trichocarpa*. Stem cross-sections were hybridized with digoxigenin-labeled antisense RNA probes for *PtrSCZ3* (left) or sense RNA probes as negative control (right). ca, cambium; pf, phloem fiber; ph, phloem; xy, xylem. Bar, 100 μm.

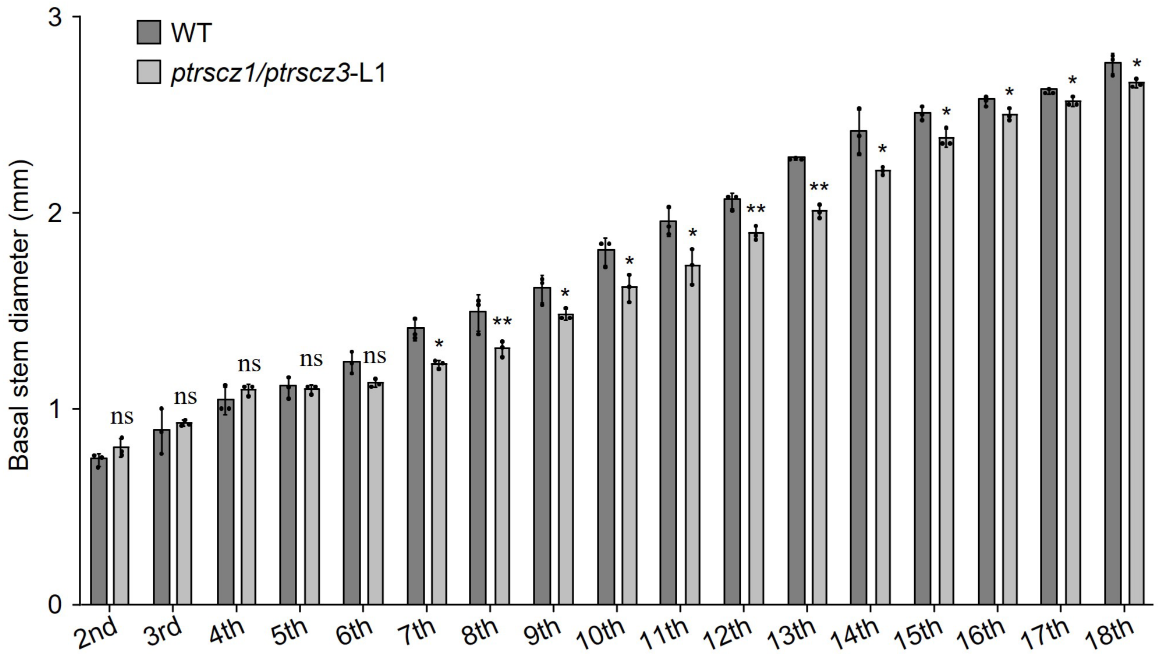

Supplementary Figure 4. The basal stem diameter of *ptrscz1/scz3* mutants. Error bars represent mean ± SE from three biological replicates. Two-tailed Student's *t*-test, * $P<0.05$, **$P<0.01$. ns, not significant differences.

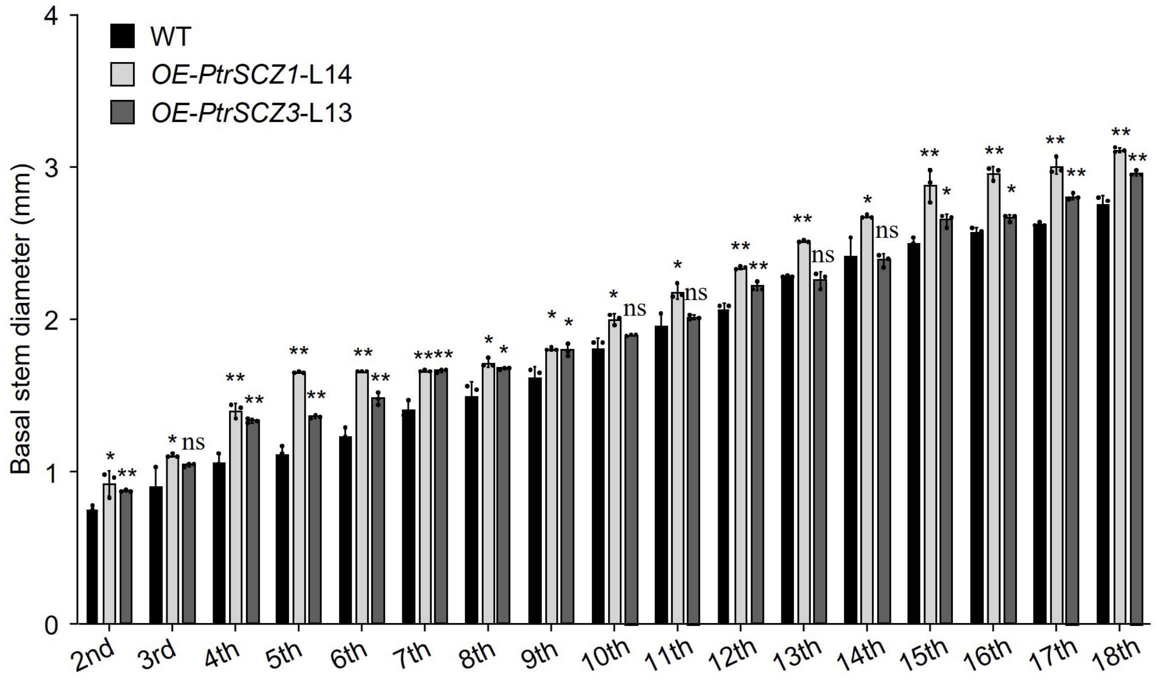

Supplementary Figure 5. The basal stem diameter of *OE-PtrSCZ1*-L14 and *OE-PtrSCZ3*-L13. Error bars represent mean ± SE from three biological replicates. Two-tailed Student's *t*-test, * *P*<0.05, ***P*<0.01. ns, not significant differences.

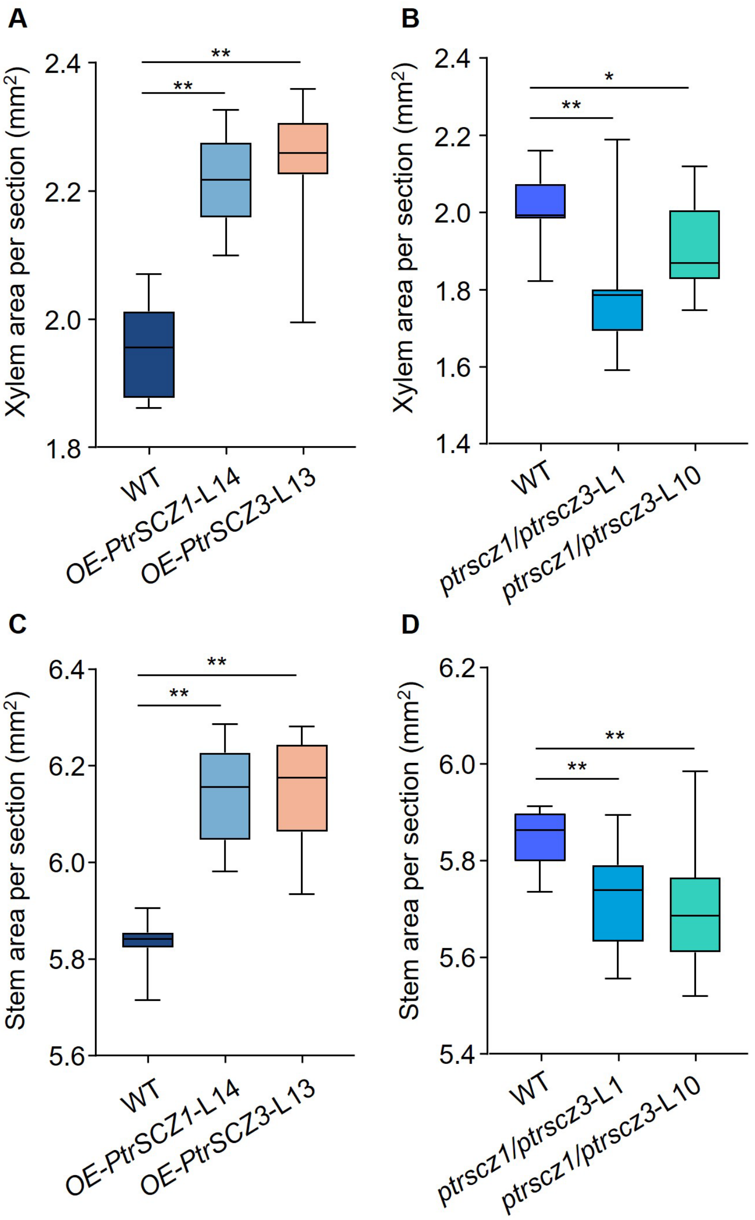

Supplementary Figure 6. Xylem and stem area of transgenic plants. (A, B) Quantitative measurements of xylem area proportion in stems among *OE-PtrSCZ1*-L14, *OE-PtrSCZ3*-L13, and WT (**A**), *ptrscz1/ptrscz3*-L1, *ptrscz1/ptrscz3*-L10, and WT (**B**). **(C, D)** Quantitative measurements of stem area among *OE-PtrSCZ1*-L14, *OE-PtrSCZ3*-L13, and WT (**C**), *ptrscz1/ptrscz3*-L1, *ptrscz1/ptrscz3*-L10, and WT (**D**). Xylem and stem areas were quantified from five cross-sections per stem internode for each biological replicate. Data from three biological replicates were analyzed. Two-tailed Student's *t*-test, * P<0.05, **P < 0.01. n=15. Box plots display median and interquartile ranges, with whiskers representing data ranges excluding outliers.

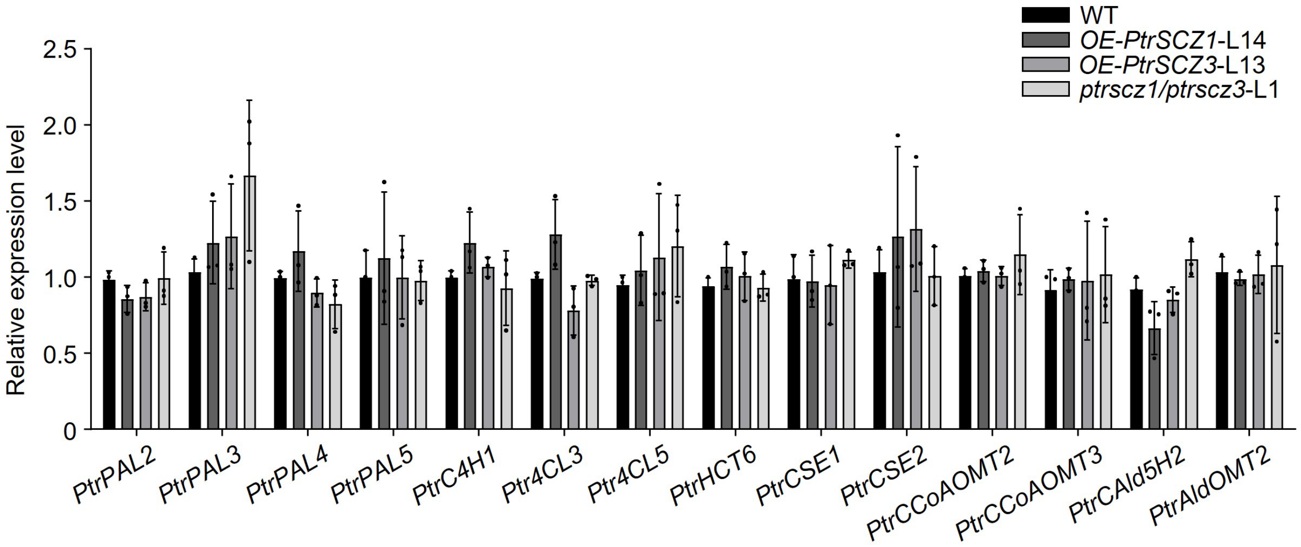

Supplementary Figure 7. Expression levels of cell wall component genes in transgenic plants. The SDX tissues from WT, *OE-PtrSCZ1*-L14, *OE-PtrSCZ3*-L13, and *ptrscz1/ptrscz3*-L1 plants were analyzed by qPCR to examine expression patterns of monolignol biosynthesis genes. Genes are arranged according to their enzymatic order in the lignin biosynthesis pathway. Error bars represent mean ± SE from three biological replicates using independent pools of *P. trichocarpa* SDX tissues. Statistical analysis revealed no significant difference.
